# Bioactivity-Guided Identification and Cell Signaling Technology to Delineate the Lactate Dehydrogenase A Inhibition Effects of *Spatholobus suberectus* on Breast Cancer

**DOI:** 10.1371/journal.pone.0056631

**Published:** 2013-02-14

**Authors:** Zhiyu Wang, Dongmei Wang, Shouwei Han, Neng Wang, Feizhi Mo, Tjing Yung Loo, Jiangang Shen, Hui Huang, Jianping Chen

**Affiliations:** 1 School of Chinese Medicine, The University of Hong Kong, Pokfulam, Hong Kong, China; 2 School of Pharmaceutical Science, Sun Yat-sen University, Guangzhou, China; 3 Guangdong Provincial Hospital of Chinese Medicine, The Second Clinical Medical Collage, University of Guangzhou Traditional Chinese Medicine, Guangzhou, China; 4 Sun Yat-sen Memorial Hospital, Sun Yat-sen University, Guangzhou, China; University of Texas Health Science Center, United States of America

## Abstract

Aerobic glycolysis is an important feature of cancer cells. In recent years, lactate dehydrogenase A (LDH-A) is emerging as a novel therapeutic target for cancer treatment. Seeking LDH-A inhibitors from natural resources has been paid much attention for drug discovery. *Spatholobus suberectus* (SS) is a common herbal medicine used in China for treating blood-stasis related diseases such as cancer. This study aims to explore the potential medicinal application of SS for LDH-A inhibition on breast cancer and to determine its bioactive compounds. We found that SS manifested apoptosis-inducing, cell cycle arresting and anti-LDH-A activities in both estrogen-dependent human MCF-7 cells and estrogen-independent MDA-MB-231 cell. Oral herbal extracts (1 g/kg/d) administration attenuated tumor growth and LDH-A expression in both breast cancer xenografts. Bioactivity-guided fractionation finally identified epigallocatechin as a key compound in SS inhibiting LDH-A activity. Further studies revealed that LDH-A plays a critical role in mediating the apoptosis-induction effects of epigallocatechin. The inhibited LDH-A activities by epigallocatechin is attributed to disassociation of Hsp90 from HIF-1α and subsequent accelerated HIF-1α proteasome degradation. *In vivo* study also demonstrated that epigallocatechin could significantly inhibit breast cancer growth, HIF-1α/LDH-A expression and trigger apoptosis without bringing toxic effects. The preclinical study thus suggests that the potential medicinal application of SS for inhibiting cancer LDH-A activity and the possibility to consider epigallocatechin as a lead compound to develop LDH-A inhibitors. Future studies of SS for chemoprevention or chemosensitization against breast cancer are thus warranted.

## Introduction

Cancer cells can be distinguished from normal cells in several hallmarks including sustaining proliferative signaling, avoiding immune destruction, resisting cell death, genome instability and disordering angiogenesis, etc [1]. One of the hallmarks is that cancer cells have a fundamentally different bioenergetic metabolism from that of non-neoplastic cells [2], [3]. In normal cells, energetic metabolism mainly relies upon the mitochondrial oxidative phosphorylation. In contrast, due to the hypoxia microenvironment and mitochondrial gene mutations, cancer cells have developed altered metabolism that predominantly produce energy by glycolysis, even in the presence of oxygen - this is known as the “Warburg Effect” [4]. Cancer glycolysis is a critical step in carcinogenesis and oncogenic activation. Targeting on glycolysis pathway has already become an important strategy for cancer diagnosis and treatment in clinic [5].

The glycolysis pathway is a series of metabolic reactions catalyzed by multiple enzymes or enzyme complexes. From the original glucose uptake to the final lactate production, the key steps involved in the process include: (1) the increasing uptake of glucose by elevated expression of Glucose transporter-1 (GLUT1) and Sodium Glucose Cotransporter-1 (SGLT1); (2) active ATP generation reaction by up-regulation of phosphoglycerate kinase (PGK) and pyruvate kinase (PK); (3) regeneration of NAD^+^ by lactate dehydrogenase (LDH); and (4) out-transport and re-uptake of lactate by monocarboxylate transporter (MCT), mainly MCT1 and MCT4 [5], [6], [7]. Most enzymes' activities in the pathway are controlled by two factors including c-myc and HIF-1α [8], [9]. Many reports have demonstrated an increased level of activities of the glycolytic enzymes in various types of tumors and cancer cell lines such as GLUT1, hexokinase, MCTs and HIF-1α [10]. In addition, silencing of these over-expressed enzymes, such as pyruvate kinase (PKM2), have been documented for inhibiting cancer cell proliferation effectively, inducing apoptosis and reversing multi-drug resistance [11], [12]. Therefore, developing novel glycolysis inhibitors is an important direction in current cancer research. Some glycolysis inhibitors such as 2-deoxy-glucose and 3-bromo-pyruvate have already been approved for clinical trials [5].

In recent years, LDH-A is also emerging as a novel cancer therapeutic target. Numerous studies have demonstrated the over-expression of LDH-A in various types of cancer including renal, breast, gastric and nasopharyngeal, etc [13], [14], [15]. Considering the important role of LDH-A in maintaining NAD^+^ regeneration, its inhibition might lead to energy production blockade in cancer cells. Several studies have found that LDH-A suppression in cancer cells result in the reactive oxygen species (ROS) burst, mitochondrial pathway apoptosis and limited tumorigenic abilities [15], [16]. LDH-A is an effective target for cancer therapy because its expression is largely confined to skeletal muscle. Moreover, human subjects with LDH-A deficiency show no apparent diseases except myoglobinuria under intense anaerobic exercise [17]. Therefore it is promising to develop novel LDH-A inhibitors. In fact, gossypol, a polyphenolic compound initially applied as male anti-fertility agent, has been demonstrated to possess the property of anti-LDH-A activity since several years ago [18]. However, the significant toxicities induced by gossypol (including cardiac arrhythmias, renal failure, muscle weakness and even paralysis) have stopped its further development in that direction. To identify a natural lead compound with less toxicity therefore becomes a focus in anti-LDH-A drug discovery.

Traditional Chinese Medicine (TCM) is particularly appreciated for cancer therapy in China. With the existence of between 250,000 to 300,000 plant species in the world, Chinese herbal medicine provides a fast track and important source for drug discovery and is becoming more and more acceptable around the world [19]. In TCM system, *Spatholobus suberectus Dunn*. (SS) is the representative herb which is believed to fight against cancer *via* modulating tumor microenvironment, such as relieving blood stasis, improving hypoxia and decreasing lactic acid accumulation, etc. Clinical and laboratory trials also demonstrated that the herb extracts could improve hematopoiesis and enhance immunity in cancer patients after they complete chemotherapy or radiotherapy [20], [21]. Some studies also indicated that the herb extracts could inhibit the proliferation of various cancer cell lines *in vitro* [22]. Chemical analysis found that SS contained flavonoids, phenolic compounds, quinones, and saponins [23], [24]. However, there still lacks strong evidence at present to support the application of SS in anti-cancer therapy. In addition, since there might exist hundreds of phytochemicals in a single herb, it is difficult to determine the actual bioactive anti-cancer compounds in SS. In recent years, target-based strategy is becoming an important tool for designing novel drugs or seeking bioactive lead compounds from natural resources. Herein, we applied the bioactivity-guided fractionation targeting on LDH-A to elucidate the active compounds in SS.

In the present study, we demonstrated the LDH-A inhibition activities of SS on breast cancer by both *in vitro* and *in vivo* assays. Meanwhile, epigallocatecin (EGC) was validated as the key compound in SS accounting for the inhibited LDH-A activity by bioactivity-guided fractionation based on LDH-A activity, LDH-A expression and apoptosis. Mechanism studies revealed that disassociation of Hsp90 from HIF-1α and subsequent accelerated HIF-1α proteasome degradation might lead to the LDH-A inhibition induced by epigallocatechin. Our study sheds novel light on the medicinal application of SS and the potential development of epigallocatechin as LDH-A inhibitors in breast cancer treatment.

## Materials and Methods

### Cell culture

Human breast cancer cell line MCF-7 and MDA-MB-231 were obtained from the American Type Culture Collection. The cells were cultivated in medium (DMEM for MCF-7; L-15 for MDA-MB-231) supplemented with 10% FBS and 1% penicillin and streptomycin at 37°C in a humidified incubator with 5% CO_2_ or without CO_2_.

### Preparation of SS aqueous extracts

SS (bought from Guang Xi Province and identified by the Institute of Botany, The Chinese Academy of Sciences) was cut into small pieces and immersed in distilled water. The mixture was treated by ultrasound for 1 hr followed by heating at 100°C for 30 mins twice. The supernatants were concentrated by rotary evaporation and kept overnight at −80°C. The frozen supernatant was then placed in the freezing dryer for 48 h to get the raw aqueous extract powder. The production ratio of SS was 10.2–11.0%. HPLC analyses of different batches of SS aqueous extracts were compared to guarantee repetitive production. The powder was dissolved in phosphate buffer solution and passed through 0.45 µm filter for later use.

### Flow cytometry analysis

For apoptosis assay, cells were seeded on 6 well plates at a density of 5×10^5^/well. After drug treatment for 48 h, cells were stained with Annexin V-Cy5 (Biovision, Mountain View, CA) for 15 mins at room temperature. The apoptotic index was determined by flow cytometry (BD Bioscience) with FlowJo software. Mitochondrial membrane potential Δψm was assessed with the fluorescent probe JC-1 (Molecular probe, Eugene, OR). The mitochondrial J-aggregated form, presenting as red fluorescence, will be assessed by flow cytometry. For superoxide (O_2_^−^) level detection, probe hydroethidine (140 µg/mL, Polysciences, Warrington, PA) was applied, which will be enzymatically dehydrogenated to form ethidium after interacting with superoxide.

### Cellular oxygen consumption

Breast cancer cells (5×10^6^) were re-suspended in 3 ml PBS supplemented with 25 mM glucose, 1 mM pyruvate and 2% BSA. Cellular respiration of normal and drug-treated cancer cells were measured by a Clark oxygen electrode (YSI, Ohio, US), which was placed in a sealed respiration chamber. All samples were assayed in triplicate for 20 mins to generate an oxygen consumption curve.

### LDH-A activity assay

Based on detection of absorption value of β-NADH at 340 nm, *in vitro* LDH-A enzymatic system was established. In a 3 ml reaction mix, the final concentrations were 100 mM sodium phosphate, 0.12 mM β-NADH, 2.3 mM pyruvate, 0.033% (W/V) bovine serum albumin and 20 µg of total protein extracts in control or drug-treated cancer cells. The value of A340 nm was continuously recorded for 5 mins at 37°C. The LDH-A activity was calculated according to the formula: Units/mg = (ΔA340 nm/min TEST - ΔA340 nm/min BLANK) * 3* df/6.22 * 0.1 * PC, where 3 is the total volume of assay, df is the dilution factor, 6.22 is the millimolar extinction coefficient of β-NADH at 340 nm, 0.1 is the volume of added protein extracts and PC is the protein concentration.

### Immunoblotting analysis

Western blotting analysis was performed as described previously [15]. Quantified protein lysates (20 µg) were resolved in SDS-PAGE gel and transferred onto PVDF membrane (Millipore, Billerica, MA). The protein blot was then blocked in 5% BSA for 2 h, washed with TBS and then incubated overnight with primary antibody at 4°C. After incubating with secondary antibody for 2 h at room temperature, Immunoreactions were visualized with ECL advance (GE Healthcare) reagents and quantified by Quantity One software.

### PCR analysis

Total RNA in control or drug treated cancer cells were extracted using TRIzol reagent (Invitrogen) and reverse transcription were carried out using first strand CDNA synthesis kit (Fermentas) according to the manufacturer's instruction. For RT-PCR analysis, Gotaq green master mix (Promega) was used to amplify the interesting genes. The primers for LDH-A and β-actin were designed as follows: LDH-A, forward primer: 5′-GGACTTGGCAGATGAACTTG-3′, reverse primer: 5′-TCAGAG AGACACCAGCAACA-3′; β-actin forward primer: 5′-CTGGGACGACATGG AGAAAA-3′, reverse primer: 5′-AAGGAAGGCTGGAAGAGTGC-3′. Real-time PCR analysis was performed using a SYBR Green kit (Fermentas) on Roche lightcycler 480 detector. The primers for HIF-1α and β-actin were designed as follows: HIF-1α, forward primer: 5′-TTTTTCAAGCA GTAGGAATTGGA-3′, reverse primer: 5′-GTGATGTAGTAGCTGCATGATCG-3′; β-actin forward primer: 5′-CCAACCGCGAGAAGATGA-3′, reverse primer: 5′-CCAGAGGCGTACAGGG ATAG-3′. Ct value was measured during the exponential amplification phase. The relative expression level (defined as fold change) of target gene was given by 2^−ΔΔCt^ and normalized to the fold.

### Bioactivity-guided fractionation and identification of bioactive anti-cancer compounds in SS

Crude aqueous extracts of SS were firstly subjected to different polar solvents to yield petroleum ether partitioned extract (PE), ethyl acetate partitioned extract (EtOAc), n-butanol partitioned extract (BuOH) and water residues (RE). Each fraction was vacuum dried and set as the same concentration (50 µg/ml) in phosphate buffer solution for later screening by Annexin V-Cy5 staining, LDH-A activity and LDH-A expression as described previously. EtOAc fraction was further separated by marcoporous resin column and chromatographied into 12 sub-fractions. The eluted solution was set as H_2_O, 20%ETOH, 40%ETOH, 60%ETOH, 80%ETOH and 95% ETOH. HPLC analysis of each sub-fraction was conducted as described previously. After second-round screening, the peaks in the candidate sub-fractions were collected by preparative HPLC column (Kromasil C18 Media, 10 nm, 5 µm, 20.0*250 mm). Through further HPLC analysis, the peaks with high purity were subjected to identification by ESI-MS (Thermo LCQ DECA XP) and NMR (BRUKER AVANCEIII 400 MHz).

### Immunoprecipitation assay

For immunoprecipitation assay, Immunoprecipitation kit-Dynabeads Protein G (Invitrogen) was applied. Control or drug treated cancer cells were lysed in RIPA buffer. Followed by centrifugation, the supernatants were collected and incubated with protein G dynabeads, which was binding to antibody (Hsp90) in advance. After incubating at room temperature for 2 h, the Dynabeads-Ab-Ag complex were washed three times with provided washing buffer and denaturized for following immuoblotting experiments.

### Establishment of LDH-A overexpression cells

The full-length cDNA of LDH-A were amplified and subcloned into the pcDNA3.1 (+) vector (invitrogen) using the EcoRI and XhoI restric enzyme sites. The primers for LDH-A were designed as follows: upstream: 5′-ATATGAATTCA TGGCAACTCTAAAGG-3′, downstream: 5′-GCGGCCTCGAGTTAAAATTGC AGCTCCT-3′. After PCR and DNA sequencing validation, the positive plasmids were co-transfected into breast cancer cells using Turbofectin8.0 and empty vector was used as a control. After 48 h of co-incubation, transfected cells were selected in primary cell cultivated medium containing 400 µg/ml geneticin. After 2 to 3 weeks, single independent clones were randomly isolated, and each individual clone was plated separately. After clonal expansion, cells from each independent clone were tested for LDH-A expression by Western blotting.

### Breast cancer xenografts

All animal studies involving animal experiments were reviewed and approved by the University of Hong Kong's Committee for Ethical Review of Research. Both MCF-7 and MDA-MB-231 cells were re-suspended at 5×10^6^ cells/100 µl in PBS and injected into the fourth mammary gland fat pad of 4–5 week-old female nude mice. In the MCF-7 xenograft, 17β-estrogen (Sigma, St. Louis, MO) was given subcutaneously at a dose of 1.5 mg per animal at three-day intervals. After the tumors reached approximately 1 cm^3^, they were cut into 5 mm^3^ pieces and then transplanted to mammary glands respectively. Mice were divided into control and drug-treated groups (6 mice per group). SS was given by oral intake at a dose of 1 g/kg/d. The identified bioactive compounds will be given by intraperitoneal injection at low and high dose per day. Tumors were measured at three-day intervals with a caliper, and tumor volume was calculated using the following formula: volume (mm^3^) = width^2^×length/2. Body weight was also monitored every three days as an indicator of the overall health of the animals. Blood from mice in SS experiment was collected in sodium heparin coated tubes for blood toxicity assay using hemocytometer (XS-800i, Sysmex, Japan). Tumor tissues were removed at the end of the experiment and subjected to immunohistochemistry study, histological analysis and western blotting analysis.

### Immunohistochemistry and TUNEL analysis

Tumor samples obtained from *in vivo* studies were rinsed in PBS and fixed in 4% paraformaldehyde/PBS. Samples were dehydrated in gradient ethanol, paraffin embedded, and sectioned (4 um). Deparaffinized sections were stained for primary antibodies. The samples were incubated overnight with biotinylated secondary antibodies. Detection was done with avidin-biotin-HRP complex (Thermo scientific, Fremont, CA) and di-aminobenzidine as chromogen. Nuclei were counterstained with hematoxylin. Antigen-positive cells were counted in six fields per tumor sample. Results are expressed as the average ± SD of tumors per group. For TUNEL analysis, sections were permeabilized with 0.1% Trition X-100 plus 0.1% sodium citrate and then incubated with 50 µl TUNEL reaction mixture (Roche) at 37°C for 60 minutes. After rinsing with PBS three times, 50 µl Converter-POD was added and the tissue cells were incubated in a humidified chamber for 30 minutes at 37°C. DAB substrate was then added, followed by counterstaining with hematoxylin. The assay included negative controls (without terminal transferase). Apoptosis was quantified by counting the number of TUNEL-positive cells in at least six non-overlapping high-power fields on each section and evaluated.

### Statistic analysis

The data were expressed as Mean±SD. A two-tailed Student's t-test was used to examine the significance of the data between groups. Statistical significance was considered when the *P* value<0.05.

## Results

### SS induces breast cancer cell apoptosis, G2/M checkpoint arrest, ROS accumulation and LDH-A inhibition

**Figure 1:**
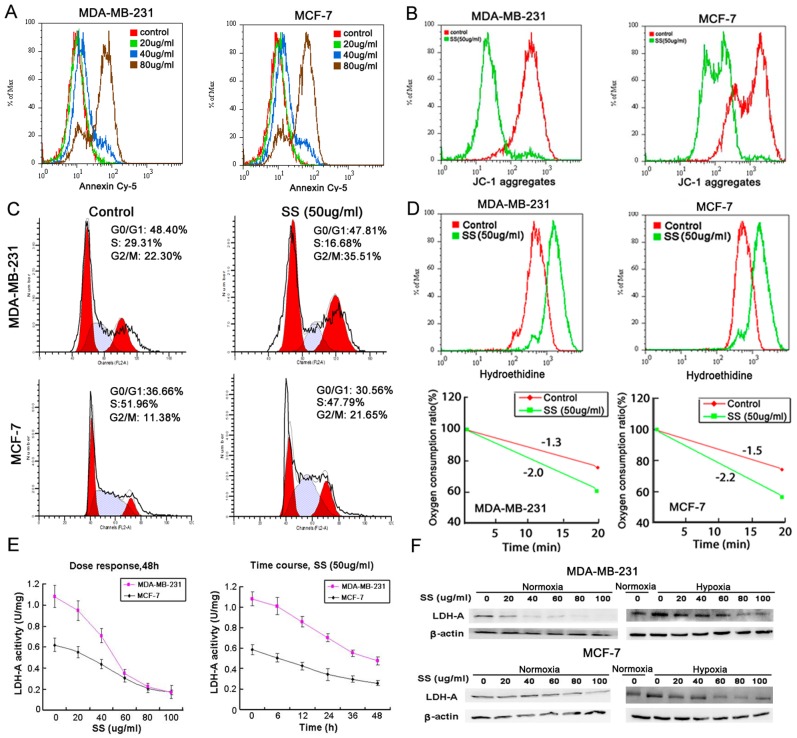
SS induces breast cancer cell apoptosis, G2/M checkpoint arrest, ROS accumulation and LDH-A inhibition. (A) Annexin-Cy5 staining results showed that SS induced both breast cancer cells apoptosis in a dose-dependent manner; (B) JC-1 staining assay indicated that mitochondrial membrane potential was decreased after SS treatment, indicating that the mitochondrial pathway apoptosis was triggered by SS; (C) Cell cycle analysis showed that after SS treatment, the G2/M checkpoint was arrested in both breast cancer cell lines, presenting as a significant increase in G2/M subpopulations; (D) Hydroethidine was applied to detect the intracellular O_2_^−^ level after SS administration by flow cytometry. The results showed that the intracellular O_2_^−^ level was increased after SS treatment for 24 h (upper panel). Clark oxygen electrode was applied to detect the oxygen consumption speed of breast cancer cells after SS treatment. The results showed that the oxygen consumption speed of both breast cancer cells was rapidly enhanced after SS administration (lower panel); (E) LDH-A activity assay showed that SS were dose- and time- dependent when suppressing the LDH-A activity; (F) Western blotting results indicated that after SS administration, the expression of LDH-A in both breast cancer cells under both normoxia and hypoxia condition were down-regulated.

Apoptosis-induction effect is an important parameter for discovery of anti-cancer agents. In our study, as shown in Figure 1A, SS could dose-dependently induce apoptosis in both MCF-7 and MDA-MB-231 cells after 48 h administration. Meanwhile, JC-1 staining also showed that the mitochondrial membrane potential Δψm was lowered after SS administration, indicating that the mitochondrial pathway apoptosis was activated (Figure 1B). Cell cycle analysis showed that in SS treated breast cancer cells, there has a significant increase in G2/M subpopulation, implying that the G2/M checkpoint was arrested by SS (Figure 1C).

As ROS has been reported as an important inductor of apoptosis and DNA damage, and O_2_^−^ accumulation was observed as an important subsequent event following LDH-A inhibition, hydroethidine was applied to detect the change of intracellular O_2_^−^ level after SS administration. As shown in Figure 1D, the fluorescence intensity of O_2_^−^ was highly elevated by SS in both breast cancer cells. In addition, oxygen consumption assay also revealed that the oxygen consumption speed was significantly sped up in SS treated cancer cells, indicating that the intracellular LDH-A activity might be suppressed by SS and the mitochondrial metabolism might be activated (Figure 1D, lower panel).

In order to see whether SS has inhibition effects on LDH-A in cancer cells, the LDH-A activity of both breast cancer cells was examined after SS administration. The results showed that SS could suppress LDH-A activity in both cancer cells in a dose- and time-dependent manner (Figure 1E). In addition, western blotting results showed that LDH-A expression was gradually inhibited with the increasing dose of SS. In order to see whether SS still possesses LDH-A inhibition effects under a hypoxic environment, a 0.5% O_2_ environment was created by importing nitrogen and 5% carbon dioxide into a sealed oxygen chamber. The LDH-A expression in both breast cancer cell was detected following SS administration for 6 h under hypoxia. The results showed that although LDH-A expression was up-regulated due to hypoxia stimulation, SS could also inhibit LDH-A expression in a dose-dependent manner (Figure 1F).

### SS inhibits breast cancer growth and LDH-A expression *in vivo*

**Figure 2:**
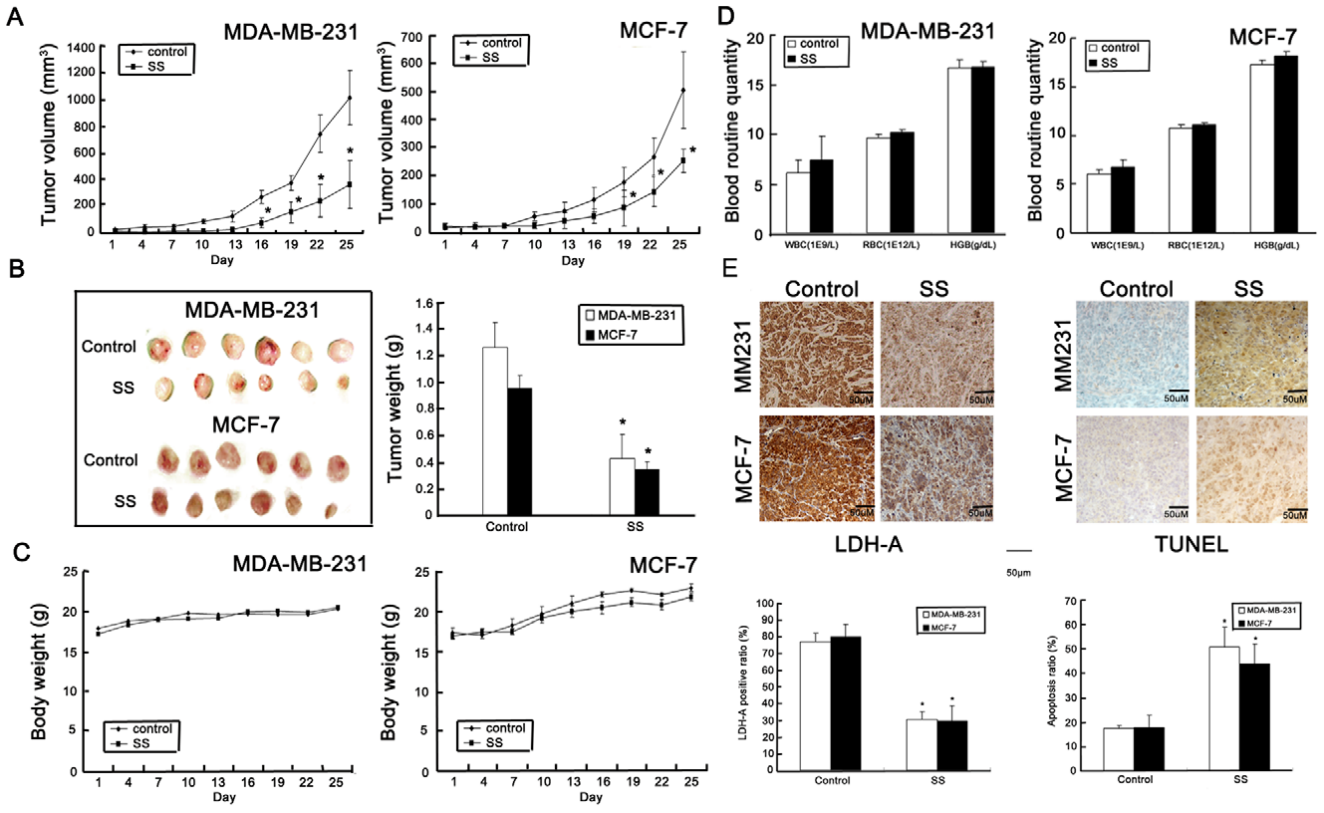
SS inhibits breast cancer growth *in vivo*. (A) Tumor growth curve of both cancer xenografts during therapy period. The results indicated that SS (1 g/kg/d, oral intake) could significantly inhibit tumor growth in both ER negative and positive breast cancers; (B) Representative tumor pictures in control and SS treated groups. The average of tumor weight in SS treated group was significantly decreased in comparison to that in the control group; (C) Body weight fluctuation influenced by SS. SS-treated group had little influences on body weight compared to the control groups; (D) Blood routine quantity assay revealed that SS has little blood toxicity effects on mice; (E) Immunohisotchemistry method was applied to detect the LDH-A expression in both control and SS treated groups. The results indicated that LDH-A expression in both SS-treated breast cancer xenografts were significantly reduced, while TUNEL analysis indicated that the apoptosis ratio in SS –treated tumor samples was significantly increased. (×100, scale bar = 50 um; All values represents as Mean±SD, n = 6, *P<0.05 *vs.* control).

To determine whether SS could inhibit cancer growth *in vivo*, human breast cancer orthotopic xenografts were built in nude mice. As shown in Figure 2A, SS significantly inhibited both cancer growth with 64.60% and 51.94% inhibition ratio on MDA-MB-231 and MCF-7 breast cancer xenografts respectively. Meanwhile, the tumor weight in SS containing group was significantly reduced in comparison with the control group (Figure 2B). In both cancer xenografts, significant body weight loss and blood toxicity did not appear in SS containing groups (Figure 2C & D). Immunohistochemistry assay showed that the LDH-A expression was suppressed in SS-treated tumor samples, while apoptosis ratio was greatly elevated (Figure 2E). All these results indicated that SS could inhibit cancer growth and LDH-A expression *in vivo*.

### Bioactivity-guided fractionation of SS

**Figure 3:**
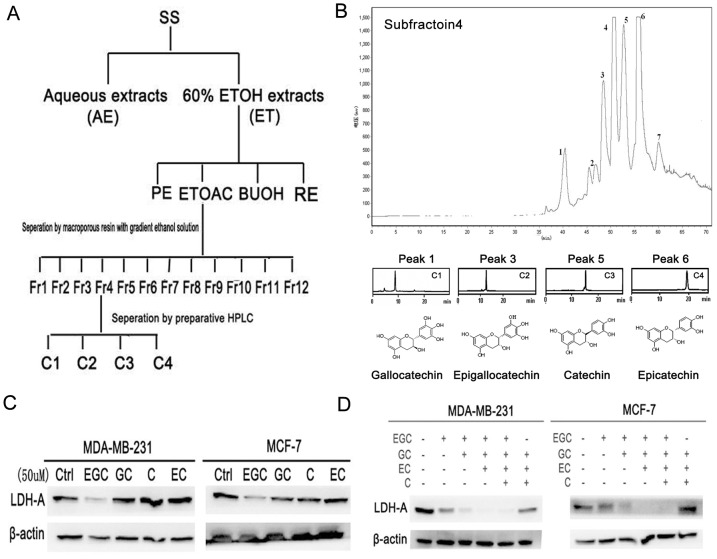
Bioactivity-guided fractionation of SS targeting on LDH-A. (A) Isolation scheme of SS. Three parameters including LDH-A activity, LDH-A expression and apoptosis were selected for bioactive compounds screening; Crude 60% ethanol extracts of SS were firstly subjected to different polar solvents to yield PE, EtOAc, BuOH and RE fractions. After the first round screening, the EtOAc fraction was further separated with macroporous resin to yield 12 subfractions. Following the second round screening, subfraction 4 was then subjected to preparative HPLC and four compounds were finally purified. (B) Preparative HPLC chromatography of subfraction 4. A total of 7 single peaks were identified and the peaks 1, 3, 5 and 6 were finally purified through preparative HPLC. After Mass and NMR identification, the four compounds were identified as gallocatechin, epigallocatechin, catechin and epicatechin; (C) Among the four compounds, EGC exhibited the highest activity in inhibiting LDH-A expression; (D) EGC played synergistic role with the other three compounds in inhibiting LDH-A expression. The four compounds were added sequentially according to their relative concentrations in SS (10 µM ∶ 25 µM ∶ 200 µM ∶ 200 µM). When the four compounds were put together, they showed the highest inhibition effects on LDH-A expression. However, when EGC was withdrawn, the inhibition effects significantly weakened, indicating that EGC might play a key role in inhibiting LDH-A activity.

In order to determine the bioactive compounds in SS, molecular-targeted screening based on LDH-A activity, LDH-A expression and apoptosis were carried out. The bioactivity-guided process was summarized in Figure 3A. Crude 60% ethanol extracts of SS were firstly subjected to different polar solvents to yield PE, EtOAc, BuOH and RE fractions. The first round screening identified EtOAc fraction as the subset with the strongest LDH-A inhibition effects. EtOAc fraction was further separated by Octadecylsilyl (ODS) column and chromatographied into 12 sub-fractions. After the second round screening, the sub-fraction 4 was finally identified as the best subset (Supplementary Figure S1). Preparative HPLC column was applied to purify bioactive compounds in sub-fraction 4. Finally a total of 7 peaks were collected. Through HPLC analysis, the peaks including 1, 3, 5 and 6 showed high purity, therefore we conducted chemical identification on them by mass and NMR. The four compounds were validated as gallocatechin (GC), epigallocatechin (EGC), catechin (C) and epicatechin (GC) (Figure 3B). Detailed structure information were listed as followings: Peak 1 (gallocatechin, GC): UV λmax: 215, 270 nm, MS: m/z 305 [M-H]-; 1H NMR (400 MHz, DMSO): δ 9.14 (s, 1H, -OH), 8.91 (s, 1H, -OH), 8.75 (s, 2H, -OH), 7.99 (s, 1H, -OH), 6.24 (s, 2H, H-2′, H-6′), 5.88 (br s, 1H, H-6), 5.69 (br s, 1H, H-8), 4.83 (d, J = 4.9 Hz, 1H, 3-OH), 4.42 (d, J = 6.9 Hz, 1H, H-2), 3.80–3.74 (m, 1H, H-3), 2.61 (dd, J = 16.0, 5.0 Hz, 1H, H-4), 2.34 (dd, J = 15.9, 7.4 Hz, 1H, H-4).13C NMR (100 MHz, DMSO) δ 156.4, 156.1, 155.2 (C-5, C-7, C-8a), 145.6 (C-3′, C-5′), 132.4 (C-4′), 129.8 (C-1′), 105.9 (C-2′, C-6′), 98.9 (C-4a), 95.0 (C-6), 93.8 (C-8), 81.0 (C-2), 66.3 (C-3), 27.4 (C-4); Peak 3 (epigallocatechin, EGC):UV λmax: 215, 271 nm, MS: m/z 305 [M-H]-, 1H-NMR (400 MHz, DMSO): δ 9.09 (s, 1H, 5-OH), 8.89 (s, 1H, 7-OH), 8.69 (s, 2H, 3′-OH, 4′-OH), 7.93 (s, 1H, 5′-OH), 6.37 (s, 2H, 2′,6′-H), 5.88 (d, J = 2.2 Hz, 1H, 6-H), 5.71 (d, J = 2.2 Hz, 1H, 8-H), 4.65 (s, 1H, 2-H), 4.60 (d, J = 4.6 Hz, 1H, 3-OH), 3.98 (m, 1H, 3-H), 2.66 (dd, J = 16.3, 4.4 Hz, 1H, 4-H), 2.45 (d, J = 3.3 Hz, 1H, 4-H). 13C NMR (100 MHz, DMSO) δ 156.5, 156.2, 155.7 (C-5, C-7, C-8a), 145.3 (C-3′, C-5′), 132.1 (C-4′), 129.7 (C-1′), 106.0 (C-2′, C-6′), 98.5 (C-4a), 95.0 (C-6), 94.1 (C-8), 78.1 (C-2), 65.0 (C-3), 28.1 (C-4). Peak 5 (catechin, C): UV λmax: 215, 276 nm, MS: m/z 289 [M-H]-, 1H-NMR (400 MHz, DMSO): δ 9.17 (s, 1H, 5-OH), 8.93 (s, 1H, 7-OH), 8.85 (s, 1H, 3′-OH), 8.81(s, 1H, 4′-OH), 6.70(m, 1H, 2′-H), 6.60 (d, J = 1.9 Hz, 1H, 5′-H), 6.58(d, J = 1.9 Hz, 1H, 6′-H),5.88 (d, J = 2.3 Hz, 1H, 6-H), 5.68 (d, J = 2.2 Hz, 1H, 8-H), 4.86 (d, J = 5.1 Hz, 1H, 3-OH), 4.47 (d, J = 7.5 Hz, 1H, 2-H), 3.82 (m, 1H, 3-H), 2.65 (dd, J = 16.1, 5.3 Hz, 1H, 4-H), 2.34 (dd, J = 16.0, 8.0 Hz, 1H, 4-H); 13C-NMR (400 MHz, DMSO): δ 156.88 (C-7), 156.59 (C-5), 155.78 (C-8a), 145.85 (C-3′, 4′), 131.01 (C-1′), 118.85 (C-6′), 115.49 (C-2′), 114.95 (C-5′), 99.47 (C-4a), 95.52 (C-6), 94.25 (C-8), 81.43 (C-2), 66.74 (C-3), 28.31 (C-4). Peak 6 (epicatechin, EC): UV λmax: 203, 278 nm MS: m/z 289 [M-H]-, 1H-NMR (400 MHz, DMSO): δ 9.13 (s, 1H, 5-OH), 8.93 (s, 1H, 7-OH)), 8.80 (s, 1H, 3′-OH), 8.72 (s, 1H, 4′-OH), 6.88 (s, 1H, 2′-H), 6.65 (m, 2H, 5′, 6′-H), 5.89 (d, J = 2.1 Hz, 1H, 6-H), 5.71 (d, J = 2.1 Hz, 1H, 8-H), 4.73 (s, 1H, 2-H), 4.68 (d, J = 2.7 Hz, 1H, 3-OH), 3.98 (m, 1H, 3-H), 2.67 (dd, J = 16.2, 3.8 Hz, 1H, 4-H), 2.45(dd, J = 16.0, 2.2 Hz, 1H, 4-H). 13C-NMR (400 MHz, DMSO): δ 156.96 (C-7), 156.66 (C-5), 156.21 (C-8a), 144.90 (C-3′, 4′), 131.04 (C-1′), 118.37 (C-6′), 118.37(C-2′), 115.32(C-5′), 98.91 (C-4a), 95.48 (C-6), 94.50 (C-8), 78.49 (C-2), 65.34 (C-3), 28.66 (C-4).The concentrations of the four compounds in the sub-fraction 4 were determined as 0.59% (GC), 1.46% (EGC), 12.36% (C) and 12.04% (EC), respectively.

Among the four compounds, EGC showed the highest LDH-A inhibition effects, while the other three did not (Figure 3C). In order to see whether EGC has synergistic effects with GC, C and EC, the four compounds were put together according to their concentration ratio in the mixture (EGC: GC: EC: C = 25 µM∶10 µM∶200 µM∶200 µM). As shown in Figure 3D, EGC showed significant synergistic effects with the other three catechins. However, when EGC was withdrawn, the inhibition effects disappear, indicating that EGC might be the key compound in SS accounting for the LDH-A inhibition effect.

### EGC induces breast cancer cells apoptosis *via* LDH-A

**Figure 4:**
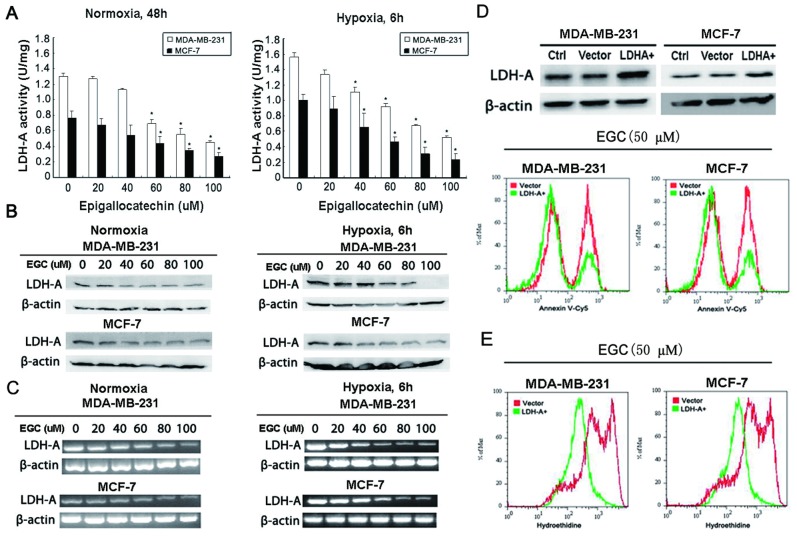
EGC inhibits breast cancer LDH-A activity and expression. (A) LDH-A activity assay was applied to detect the effects of EGC on LDH-A activity in both breast cancer cell lines. The results indicated that EGC could dose- and time-dependently suppressed the LDH-A activity (All values represents as Mean± SD, n = 3, *P<0.05 *vs.* control); (B) The expression of LDH-A was also validated by Western blotting after EGC administration. The results showed that SS could inhibit LDH-A expression under both normoxia and hypoxia condition; (C) RT-PCR method was utilized to detect the level changes of LDH-A mRNA after EGC treatment. The results revealed that LDH-A mRNA levels were also down-regulated with increasing dose of EGC under both normoxia and hypoxia conditions; (D) The full length cDNA of LDH-A were amplified and subcloned into the pcDNA 3.1(+) vector. The over-expression of LDH-A was validated by Western blotting (upper panel). Annexin V-Cy5 staining analysis demonstrated that the EGC- induced apoptosis was reduced in LDH-A over-expression breast cancer cells; (E) Hydroethidine staining assay also revealed that in LDH-A over-expression breast cancer cells, the EGC-induced ROS elevation was also significantly eliminated.

To validate the effects of EGC on LDH-A activity and expression, *in vitro* enzymatic activity assay and western blotting were applied together to detect the EGC effects on LDH-A. The results showed that the LDH-A activity and expression in both breast cancer cells were down-regulated in a dose-dependent manner after EGC treatment under both normoxic and hypoxic condition (Figure 4A & B). RT-PCR results revealed that EGC also inhibited LDH-A mRNA levels, indicating that the decreased LDH-A activity might happen at the transcriptional level (Figure 4C).

In order to validate the critical role of LDH-A in EGC-induced apoptosis, we compared the apoptotic events in parental and LDH-A over-expression cancer cells. The results showed that LDH-A over-expression reduced apoptosis induced by EGC detecting by Annexin V-Cy5 staining (Figure 4 D). Meanwhile, the high-elevated O_2_^−^ level induced by EGC was also down-regualted in LDH-A over-expression cancer cells, implying that LDH-A might be a key molecular target in the EGC-induced apoptosis (Figure 4E).

### EGC promotes HIF-1α proteasome degradation *via* HSP90

**Figure 5:**
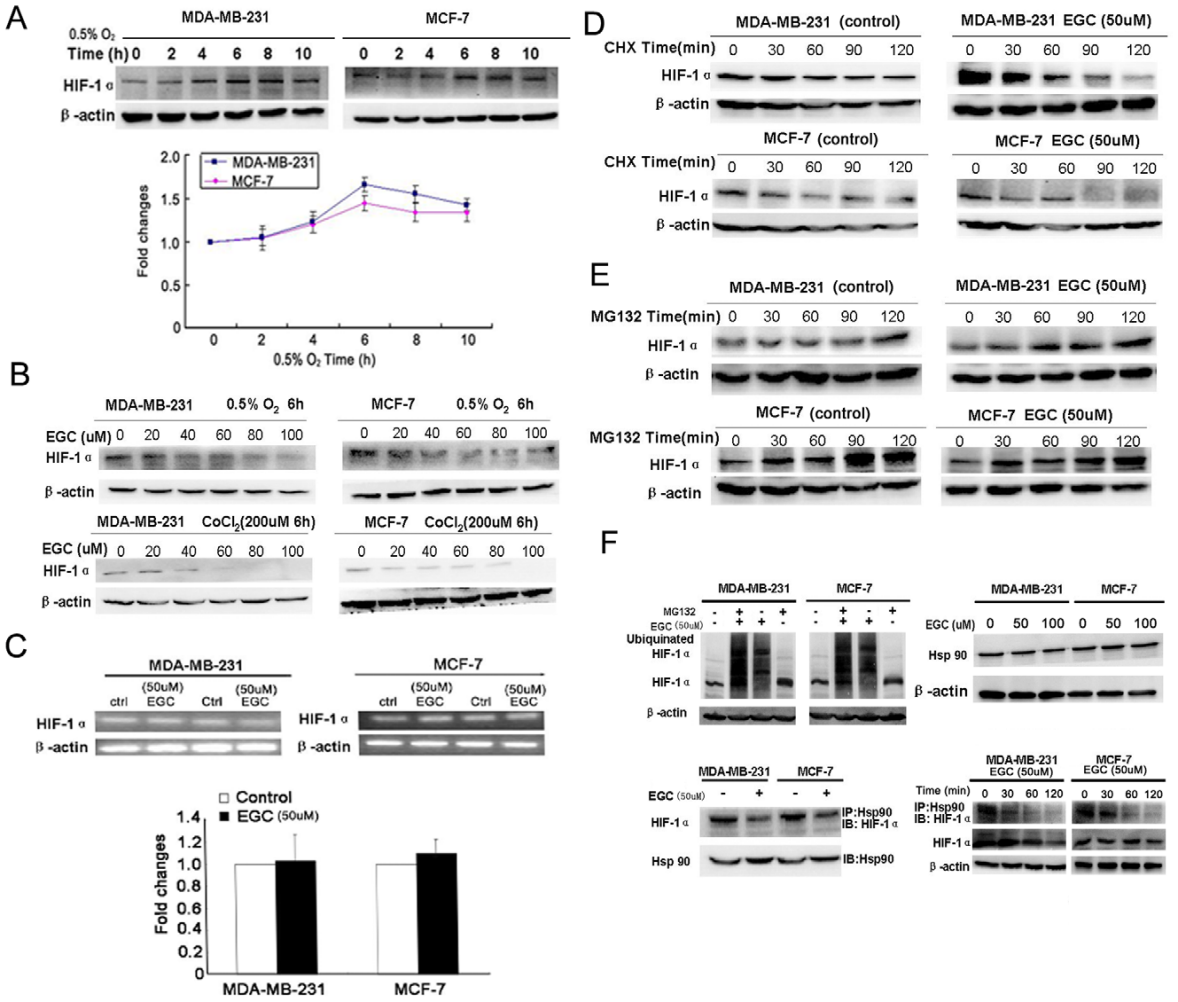
EGC promotes HIF-1α proteasome pathway degradation. (A) Expression curve of HIF-1α under hypoxia. The results showed that the expression level of HIF-1α reached the peak at the 6^th^ hour, but had a slight decrease after 10 hours; (B) EGC inhibited HIF-1α expression in a dose-dependent manner in both breast cancer cells under both hypoxia condition and hypoxia mimic created by cobalt chloride; (C) Real-time PCR analysis was applied to detect the influences of EGC on HIF-1α mRNA levels. The results showed that there were little changes on HIF-1α mRNA level after EGC administration; (D) Cychloheximide (CHX) was administrated on breast cancer cells to inhibit protein synthesis. The expression of HIF-1α in both control and EGC treated cancer cells were validated by western blotting. The results showed that the HIF-1α degradation speed was much faster in EGC treated groups than in control groups, indicating that EGC might promote HIF-1α degradation; (E) MG132 was administrated on breast cancer cells to block the proteasome activity. The levels of HIF-1α were determined after MG132 treatment in both control and EGC treated groups. The results indicated that the accumulation quantities of HIF-1α were similar in both control and EGC treated groups, indicating that proteasome degradation was the main pathway accounting for decreased HIF-1α expression; (F) The ubiquinated HIF-1α level in both control and EGC treated group were detected by Western blotting. The results showed that EGC increased the expression level of ubiquinated HIF-1α in both cancer cells, indicating that EGC promoted the HIF-1α proteasome degradation pathway; Meanwhile, the interaction between HIF-1α and Hsp90 were validated by immunoblotting and immunoprecipitation assay. The results indicated that EGC brought little influences on Hsp90 expression. In EGC treated cell samples, the levels of HIF-1α binding to Hsp90 were decreased in comparison to control groups. Meanwhile, the dissociation of HIF-1α from Hsp90 was demonstrated to be an early event happed before downrgulation of HIF-1α induced by EGC, implying that EGC promotes HIF-1α degradation *via* interfering with the interaction between Hsp90 and HIF-1α. (All values represented as Mean± SD, n = 3, *P<0.05 *vs.* control).

HIF-1α is reported as an important regulator of LDH-A. Since HIF-1α has the strongest expression at the 6^th^ hour under hypoxia, we selected the 6^th^ hour for HIF-1α—EGC interaction study (Figure 5A). The results showed that EGC inhibited HIF-1α expression under both hypoxic and hypoxic mimic microenvironment created by CoCl_2_ (Figure 5 B). In order to validate whether EGC had influences on HIF-1α mRNA expression, Q-PCR analysis was carried out. The results showed that there was little expression changes on HIF-1α mRNA levels after 50 µM EGC treatment (Figure 5C).

Besides transcription regulation, HIF-1α protein level was also influenced by post-translational processes. PI3K/Akt/mTOR and proteasome degradation pathway were reported as the most important regulators. Since previous study demonstrated that there was little expression of phosphor-Akt in breast cancer cell lines MCF-7 and MDA-MB-231 [25], we therefore focused on the HIF-1α proteaosme degradation pathway. Cycloheximide, a protein synthesis inhibitor, was firstly added with or without EGC to treat breast cancer cells. The results showed that in both breast cancer cells, the HIF-1α degradation speed was much faster in the EGC treated group than that in the control groups, indicating that the proteasome degradation pathway might be activated (Figure 5D). In order to validate the role of proteasome degradation in HIF-1α down-regulation caused by EGC, the proteasome inhibitor MG132 was then administrated to breast cancer cells. The results showed that the HIF-1α accumulation level was similar in EGC treated or untreated groups, indicating that the accelerated proteasome degradation pathway was mainly accouting for HIF-1α down-regulation induced by EGC (Figure 5E). Meanwhile, the increased ubiquinated- HIF-1α in EGC treated samples also supported that EGC promoted the HIF-1α proteasome degradation (Figure 5F).

HIF-1α belongs to the Per-ARNT-Sim basic helix-loop-helix family and interacts with Hsp90. It is well demonstrated that binding with Hsp90 can enhance HIF-1α stability and prevent it from degradation [26], [27]. The results from immunoblotting assays firstly demonstrated that EGC brought little influences on Hsp90 expression in both breast cancer cells. Further immunoprecipitation assay showed that EGC inhibited binding of HIF-1α to Hsp90. Meanwhile, the disassociation of HIF-1α from Hsp90 was demonstrated as an early event happened as early as 30 mins after EGC administration. However, the downregualtion of total HIF-1α was shown to happen at 2 h after EGC treatment, suggesting that EGC promotes HIF-1α degradation *via* interfering with the interaction between Hsp90 and HIF-1α (Figure 5F).

### EGC inhibits breast cancer growth and LDH-A expression *in vivo*

**Figure 6:**
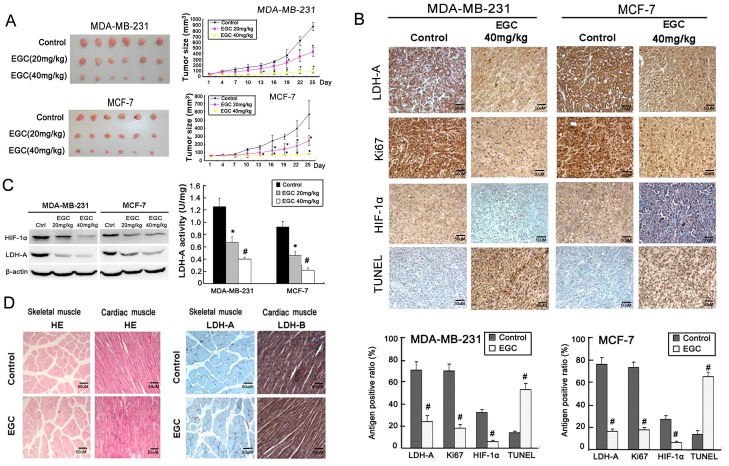
EGC inhibits breast cancer growth and LDH-A expression *in vivo*. (A) In MDA-MB-231 and MCF-7 breast cancer xenografts, EGC (20 mg/kg/d and 40 mg/kg/d) dose-dependently inhibited breast cancer growth, but had little influences on mice body weight; (B) The expression of LDH-A, Ki67 and HIF-1α in both control and EGC treated tumor samples were detected by immunohistochemistry. The results showed that in both breast cancer xenografts, the expression levels of LDH-A, Ki67 and HIF-1α were decreased in comparison to that in control groups, while apoptosis ratio was elevated in EGC-treated tumor samples detected by TUNEL method; (C) Western blotting analysis showed that the expression of HIF-1α and LDH-A in EGC-treated tumor samples were significantly decreased in comparison with control groups (left panel). The LDH-A activity in control and EGC-treated tumor samples were determined. The results showed that there was a significant reduction in LDH-A activity in EGC-treated tumor samples (right panel); (D) HE staining and immunohistochemistry assay indicated that EGC had little influences on the morphology and LDH expression in muscle and heart (×100, scale bar = 50 um; All values represents as Mean± SD, n = 6, *P<0.05 *vs.* control).

To determine whether or not EGC could inhibit HIF-1α/LDH-A expression *in vivo*, human breast cancer xenografts were built. As shown in Figure 6 A, EGC administration significantly limited cancer growth in a dose-dependent manner. Immunohistochemistry assay showed that the LDH-A, HIF-1α and Ki67 expression were all inhibited in EGC-treated tumor samples, while the apoptosis ratio was significantly elevated (Figure 6 B). Western blotting assay further demonstrated that both LDH-A and HIF-1α expression were down-regulated in EGC-treated tumor samples, which is consistent with immunohistochemistry results (Figure 6C, left panel). In addition, LDH-A enzymatic assay also revealed that the LDH-A activity of EGC-treated tumor samples was significantly decreased in comparison to that of the control group (Figure 6C, right panel). In order to see whether EGC had inhibition effects on LDH expression in other tissues and organs, we selected skeletal muscle and heart tissues, the two typical LDH expression places, to detect the influences. The results showed that EGC brought little influences on LDH expression and morphology of these tissues, indicating that EGC had little toxic effects (Figure 6D).

## Discussion

Targeting on hypoxia and glycolytic related genes has become an important strategy for cancer therapy and drug development. LDH-A is a hypoxia response gene and its over-expression has been demonstrated in numerous types of cancer. LDH-A silencing was reported to induce cancer cells death by recent studies. Given that LDH-A inhibition has little toxic effects on normal human tissues, it might be an ideal therapeutic target for cancer treatment and it is of great interest to develop its natural inhibitors from plants. Through long-history clinical application, TCM has well been accepted by pharmacologists and oncologists as a valuable database to screen bioactive compounds for novel drug discovery. In TCM system, SS is believed to modulate tumor hypoxia microenvironment and has long been recommended for cancer therapy. However, the anti-cancer mechanisms of SS and its bioactive compounds still remain unclear.

In our present study, we firstly demonstrated the inhibition effects of SS on LDH-A activity using *in vitro* and *in vivo* models. Previous study has demonstrated that in LDH-A knockdown breast cancer cells, the oxygen consumption was demonstrated greatly speeded up [15]. As the OXPHOS chains in the mitochondrion of cancer cells are mutated in comparison with those of normal cells, the OXPHOS chain activation will make more electrons leak and form reactive oxygen species after combining with oxygen, which finally destroy the mitochodnrial membrane and induce mitochondrial pathway apoptosis. LDH-A was therefore considered as an upsteam factor accounting for ROS burst and mitochondrial pathway apoptosis [16]. We firstly detected the apoptosis and intracellular ROS level following SS treatment. The results showed that the mitochondrial pathway apoptosis was triggered after SS administration for 48 h, accompanying with elevated ROS level and accelerated oxygen consumption. All these results indicated that the LDH-A activity might be inhibited by SS. The results from enzymatic reaction system and Western blotting revealed that SS could inhibit both LDH-A activity and expression in a time- and dose- dependent manner. Interestingly, SS interestingly exhibited stronger inhibitory effects on the activity and expression of LDH-A in MDA-MB-231 cells than it did in MCF-7 cells, implying that ER negative cells might be more sensitive to SS than ER positive ones. Actually, the relation between LDH and ER has been studied previously by other scientists, they found that the mean value of LDH in ER+PR+ tumors was lower than in the ER-PR- subset regardless of menopausal status [28]. In our study, we also found that the LDH-A activity in MDA-MB-231 cells was greatly higher than that of MCF-7. However, it can not say that ER could negatively regulate LDH-A expression. Some studies also found that estrogen could positively control LDH expression in ER-positive cells [29]. Therefore the regulatory network between ER and LDH seems to be complicated, which might be controlled by multiple factors. Whether ER will affect the activity of SS on LDH-A might need further investigation. Given the hypoxic microenvironment of cancer *in vivo*, we placed cancer cells in a hypoxic chamber to validate whether SS could also result in a similar LDH-A suppression or not. The results showed that a similar LDH-A inhibition effect was observed. These results indicated that SS might reach a similar anti-LDH-A effect *in vivo*. *In vivo* study showed that SS could significantly inhibit breast cancer growth. Immunohistochemistry results further showed that the expression of LDH-A was significantly suppressed in groups treated tumor samples, while the apoptosis ratio was elevated, implying that SS could also inhibit cancer cell proliferation, LDH-A expression and trigger apoptosis *in vivo*. It is of great interest to seek the bioactive compounds in SS targeting on LDH-A.

Bioactivity-guided fractionation has been considered as a novel strategy leading to drug discovery in recent years [30]. LDH-A activity and expression were both suppressed by SS while LDH-A expression was closely correlated to apoptosis. These three parameters including LDH-A activity, LDH-A expression and apoptosis were selected for bioactivity screening. Finally a sub-fraction enriched in catechins was validated with the strongest LDH-A inhibition and apoptosis induction effect. We further isolated four compounds in the sub-fraction identified as GC, EGC, C and EC. Among them, EGC exhibited the highest LDH-A inhibition effect and GC, C, EC showed synergistic LDH-A suppression effects with EGC. For the synergistic effect, since catechins are considered as basic units of procyanidins, which are oligomeric compounds formed by carbon-carbon bond interactions between C, EC, EGC or other their derivatives. Therefore we applied vanillin/H2SO4 assay to detect the procyanidins concentrations in SS. The results showed that procyanidins concentrations in ethanol extracts of SS is 51.09±0.37%, indicating that the catechins could form procyanidins in SS, therefore exhibiting a synergistic effects when played together. Catechins are defined as flavonoids compounds, and widely distributed in various plants and fruits, such as green tea, grape seeds, strawberries, etc [31], [32]. Numerous epidemiological and laboratory studies have indicated that catechins intake is beneficial for cancer prevention, delaying cancer onset and eliminating carcinogens [33], [34]. The anti-cancer molecular mechanisms of catechins are validated to occur in multiple pathways including both direct and indirect interaction such as apoptosis triggering, cell cycle arrest, angiogenesis inhibition, ROS induction, tyrosine kinases inhibition and interruption of survival signaling pathways, etc. Our study for the first time revealed that catechins could also inhibit LDH-A activity in cancer cells.

In comparison to EGCG, little attention has been paid to the anti-cancer effects and mechanisms of EGC, a more popular poylphenolic compound in natural plants. Actually some studies demonstrated that EGC has similar anti-cancer effects in comparison with EGCG [35], [36]. In the present study, we have shown that EGC inhibited LDH-A expression and activity under both normoxia and hypoxia conditions. The administration concentration of EGC is equivalent to the reported EGCG effective dose. Meanwhile, LDH-A over-expression significantly reduced EGC-induced apoptosis, indicating that LDH-A might be a critical molecule in mediating anti-cancer effects of EGC. HIF-1α is the most important transcriptional regulator of LDH-A. HIF-1α activates LDH-A expression by binding to the hypoxia response element in the LDH-A promoter region. Herein, we firstly found that EGC could inhibit HIF-1α expression under both hypoxia and hypoxia mimic conditions, while there are little changes on its mRNA expression level, indicating that protein degradation might be a main mechanism accounting for HIF-1α down-regulation. The following experiments revealed that EGC sped up HIF-1α proteasome degradation. Hsp90, a chaperone protein, has been shown to stabilize HIF-1α [37], [38]. EGC was found to inhibit the binding of HIF-1α to Hsp90. Meanwhile, the disassociation of HIF-1α from HSP90 was demonstrated to be an early event before HIF-1α degradation induced by EGC, suggesting that EGC promotes HIF-1α degradation by interfering with the interaction between HIF-1α and Hsp90. Besides protein degradation, multiple cellular signaling pathways were also involved in the regulation of HIF-1α stabilization and transactivation, such as PI3K/Akt pathways [39]. However, there is little phosphor-Akt expression in both breast cancer cells demonstrated by previous studies [25]. Therefore, whether or not EGC can interrupt the PI3K/Akt signaling pathway might need further study on other cancer models.

In consistent with *in vitro* findings, *in vivo* study also revealed that EGC could dose-dependently inhibit tumor growth on both cancer xenografts without bringing significant toxic effects such as body weight loss. The expression of HIF-1α, LDH-A and Ki67 were all down-regulated, while apoptosis ratio was elevated in EGC-treated tumor samples, implying that EGC also interrupted the HIF-1α/LDH-A pathway *in vivo*.

Tumor energy supply is critical for maintaining its proliferation, angiogenesis and metastasis. Glycolysis inhibition has been considered as an important strategy for cancer therapy. In this study, we have shown that SS possessed significant anti-cancer effects *via* LDH-A inhibition both *in vitro* and *in vivo*. EGC was validated as the key compound in SS by targeting on LDH-A, the mechanism of which is closely correlated to accelerated HIF-1α proteasome degradation. These results indicated that SS might be used as an alternative treatment for cancer patients. Meanwhile, our results also benefit future design of LDH-A inhibitors based on EGC structure. Meanwhile, more in-depth *in vitro* and *in vivo* studies are further required to look into the synergistic effects of EGC with traditional chemotherapy or radiotherapy.

## Supporting Information

Figure S1 **Bioactivity-guided screening of SS targeting on LDH-A.** (A) First round screening. The aqueous extracts (AE), 60% ethanol extracts (ET) and fractions extracted by petroleum ether (PE), ethyl acetate (EtOAc), n-butanol (BUOH) and water residues (RE) of SS were subjected to screening assays including LDH-A activity, apoptosis and LDH-A expression. The results indicated that the EtOAc fractions of SS exhibited the highest inhibitory ratio on LDH-A activity and expression, accompanying with the highest apoptosis ratio; (B) 12 subfractions from EtOAc fractions were subjected into the second round screening. The results showed that the subfraction 4 possess the highest activity in inhibiting LDH-A activity, expression and inducing apoptosis; (C) Among the four compounds purified from subfraction 4, EGC showed the highest apoptosis-induction and LDH-A inhibitory effects.(All values represents as Mean± SD, n = 3, *P<0.05 vs. control).
(TIF)
