# Supplementary figures and images for: Bioactivity-Guided Identification and Cell Signaling Technology to Delineate the Lactate Dehydrogenase A Inhibition Effects of Spatholobus suberectus on Breast Cancer
Source: PLoS One. 2013 Feb 14;8(2):e56631. doi: 10.1371/journal.pone.0056631 (PMC3572989; doi:10.1371/journal.pone.0056631)

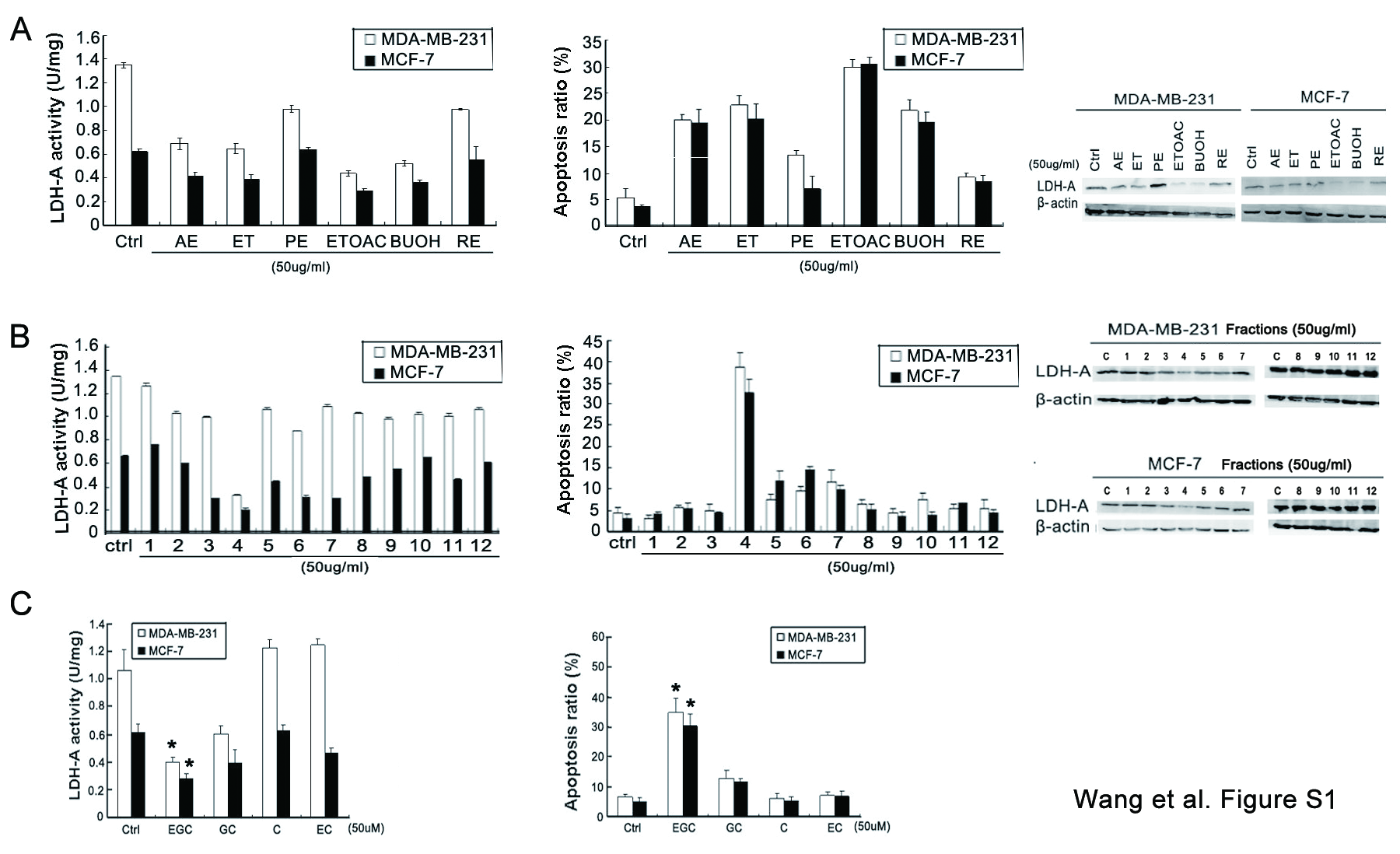

Supplement: Figure S1 — Bioactivity-guided screening of SS targeting on LDH-A. (A) First round screening. The aqueous extracts (AE), 60% ethanol extracts (ET) and fractions extracted by petroleum ether (PE), ethyl acetate (EtOAc), n-butanol (BUOH) and water residues (RE) of SS were subjected to screening assays including LDH-A activity, apoptosis and LDH-A expression. The results indicated that the EtOAc fractions of SS exhibited the highest inhibitory ratio on LDH-A activity and expression, accompanying with the highest apoptosis ratio; (B) 12 subfractions from EtOAc fractions were subjected into the second round screening. The results showed that the subfraction 4 possess the highest activity in inhibiting LDH-A activity, expression and inducing apoptosis; (C) Among the four compounds purified from subfraction 4, EGC showed the highest apoptosis-induction and LDH-A inhibitory effects.(All values represents as Mean± SD, n = 3, *P<0.05 vs. control). (TIF) [file pone.0056631.s001.tif]
